# Characteristics, treatments, in-hospital and long-term outcomes among inpatients with acute exacerbation of chronic obstructive pulmonary disease in China: sex differences in a large cohort study

**DOI:** 10.1186/s12890-024-02948-4

**Published:** 2024-03-11

**Authors:** Jiarui Zhang, Qun Yi, Chen Zhou, Yuanming Luo, Hailong Wei, Huiqing Ge, Huiguo Liu, Jianchu Zhang, Xianhua Li, Xiufang Xie, Pinhua Pan, Mengqiu Yi, Lina Cheng, Hui Zhou, Liang Liu, Adila Aili, Yu Liu, Lige Peng, Jiaqi Pu, Haixia Zhou

**Affiliations:** 1 Department of Respiratory and Critical Care Medicine, West China Hospital, Sichuan University, Guo-xue-xiang 37#, Wuhou District, 610041 Chengdu, Sichuan Province China; 2 Sichuan Cancer Hospital, University of Electronic Science and Technology of China, Chengdu, Sichuan Province China; 3 West China School of Medicine, West China Hospital, Sichuan University, Chengdu, Sichuan Province China; 4 State Key Laboratory of Respiratory Disease, Guangzhou Medical University, Guangzhou, Guangdong Province China; 5 Department of Respiratory and Critical Care Medicine, People’s Hospital of Leshan, Leshan, Sichuan Province China; 6 Department of Respiratory and Critical Care Medicine, Sir Run Run Shaw Hospital, Zhejiang University School of Medicine, Hangzhou, Zhejiang Province China; 7 Department of Respiratory and Critical Care Medicine, Tongji Hospital, Tongji Medical College, Huazhong University of Science and Technology, Wuhan, Hubei Province China; 8 Department of Respiratory and Critical Care Medicine, Union Hospital, Tongji Medical College, Huazhong University of Science and Technology, Wuhan, Hubei Province China; 9 Department of Respiratory and Critical Care Medicine, the First People’s Hospital of Neijiang City, Neijiang, Sichuan Province China; 10 Department of Respiratory and Critical Care Medicine, Xiangya Hospital, Central South University, Changsha, Hunan Province China; 11 Department of Emergency, First People’s Hospital of Jiujiang, Jiujiang, Jiangxi Province China; 12 Department of Respiratory and Critical Care Medicine, The Affiliated Hospital of Chengdu University, Chengdu, Sichuan Province China

**Keywords:** Acute exacerbation of chronic obstructive pulmonary disease, Sex differences, Characteristics, Treatments, In-hospital mortality

## Abstract

**Background:**

Data related to the characteristics, treatments and clinical outcomes of acute exacerbation of chronic obstructive pulmonary disease (AECOPD) patients in China are limited, and sex differences are still a neglected topic.

**Methods:**

The patients hospitalized for AECOPD were prospectively enrolled from ten medical centers in China between September 2017 and July 2021. Patients from some centers received follow-up for 3 years. Data regarding the characteristics, treatments and in-hospital and long-term clinical outcomes from male and female AECOPD patients included in the cohort were analyzed and compared.

**Results:**

In total, 14,007 patients with AECOPD were included in the study, and 11,020 (78.7%) were males. Compared with males, female patients were older (74.02 ± 10.79 vs. 71.86 ± 10.23 years, *P* < 0.001), and had more comorbidities (2.22 ± 1.64 vs. 1.73 ± 1.56, *P* < 0.001), a higher frequency of altered mental status (5.0% vs. 2.9%, *P* < 0.001), lower diastolic blood pressure (78.04 ± 12.96 vs. 79.04 ± 12.47 mmHg, *P* < 0.001). In addition, there were also significant sex differences in a range of laboratory and radiographic findings. Females were more likely to receive antibiotics, high levels of respiratory support and ICU admission than males. The in-hospital and 3-year mortality were not significantly different between males and females (1.4% vs. 1.5%, *P* = 0.711; 35.3% vs. 31.4%, *P* = 0.058), while female smokers with AECOPD had higher in-hospital mortality than male smokers (3.3% vs. 1.2%, *P* = 0.002) and male smokers exhibited a trend toward higher 3-year mortality compared to female smokers (40.7% vs. 33.1%, *P* = 0.146).

**Conclusions:**

In AECOPD inpatients, females and males had similar in-hospital and long-term survival despite some sex differences in clinical characteristics and treatments, but female smokers had significantly worse in-hospital outcomes than male smokers.

**Clinical Trial Registration:**

Retrospectively registered, registration number is ChiCTR2100044625, date of registration 21/03/2021. URL: http://www.chictr.org.cn/showproj.aspx?proj=121626.

**Supplementary Information:**

The online version contains supplementary material available at 10.1186/s12890-024-02948-4.

## Background

Chronic obstructive pulmonary disease (COPD) is characterized by persistent respiratory symptoms and progressive airflow obstruction, which remains a major cause of morbidity, mortality, and healthcare use worldwide [1, 2]. COPD has a prevalence of 10.1% and affects close to 400 million people worldwide [1, 3]. In China, COPD was the third leading cause of mortality and accounted for approximately 68 deaths per 100 000 population in 2017 [4]. Acute exacerbation of COPD (AECOPD) is defined as acute, abnormal deterioration of respiratory symptoms, resulting in the need for more radical treatments [5]. It has been reported that 22–40% of patients with COPD experience at least one moderate or severe exacerbation every year, while 9–16% experience more than one [6]. AECOPD has a negative impact on quality of life, accelerates lung function decline and can result in an increased risk of subsequent cardiovascular events and thromboembolic events [7–10]. Additionally, AECOPD is associated with significant hospitalization and mortality, as well as health and socioeconomic burden [6].

Patients with AECOPD have specific clinical characteristics, including more serious respiratory symptoms, worse clinical outcomes, and higher medication expenditure [11]. It is imperative to identify the characteristics and clinical outcomes to optimize clinical management and improve prognosis. Several studies have explored the characteristics, clinical outcomes and economic burden among AECOPD patients [12–15]. However, there are limited available data regarding this topic in China. Although COPD is more common among males, its prevalence is rapidly increasing among females [16]. Some studies have reported both long-term physiologic and quality-of-life differences between males and females with stable COPD [17, 18]. However, none have focused on gender-related differences during the treatments of AECOPD.

The present study provides an overview of the characteristics, treatments, in-hospital and long-term clinical outcomes among inpatients with AECOPD by using a larger longitudinal cohort from 10 hospitals in China. We additionally aimed to elucidate more clearly sex differences in patients hospitalized for AECOPD.

## Patients and methods

### Study design and participants

The MAGNET AECOPD (MAnaGement aNd advErse ouTcomes in inpatients with acute exacerbation of COPD) Registry study was a prospective, noninterventional, multicenter, real-world cohort study. Patients admitted into hospital for AECOPD was consecutively enrolled from 10 medical centers in China between September 2017 and July 2021. The admission and treatment of patients were at the discretion of the attending physicians. The major aims of the MAGNET AECOPD study were to investigate the characteristics, treatments and adverse outcomes (including intensive treatments usage; in-hospital venous thromboembolism (VTE); short- and long-term mortality, readmission, etc.) of inpatients with AECOPD and to establish and validate early warning models of these adverse outcomes, as described previously [19]. The diagnosis of AECOPD was based on the following criteria: (1) a history of COPD, including (i) exposure to risk factors (e.g., tobacco smoking, specific environment exposure); (ii) long-term dyspnea, chronic cough, or sputum production; (iii) postbronchodilator spirometry testing showing a forced expiratory volume in 1 s (FEV_1_)/forced vital capacity (FVC) ratio less than 70%; and (2) an acute worsening of respiratory symptoms resulting in additional therapy. This study was approved by the Ethics Committee of West China Hospital of Sichuan University, and written informed consent was obtained from participants.

### Data collection

A standardized case report form was completed for every enrolled patient, including baseline demographics, comorbidities, symptoms and signs on admission, laboratory and radiologic findings, treatments and in-hospital outcomes. Smoking status was dichotomized into smokers and non-smokers. Smokers referred to former or current smokers with at least 10 pack-years of smoking history. In terms of treatments, we evaluated the use of inhaled/nebulized bronchodilators, inhaled/nebulized corticosteroids, systemic corticosteroids (oral or intravenous), antibiotics, and theophylline drugs during hospitalization. We also recorded respiratory support usage including noninvasive mechanical ventilation and invasive mechanical ventilation. The enrolled individuals from 2 medical centers were followed up for 3 years by telephone, outpatient visits, or rehospitalization when necessary. The variables collected in MAGNET AECOPD study are shown in Supplementary Table 1.

### Clinical outcomes

We determined in-hospital and long-term outcomes, including all-cause in-hospital mortality, discharge against medical advice, clinical improvement at discharge, intensive care unit (ICU) admission, length of stay (LOS), total hospitalization expenses and 3-year mortality among inpatients with AECOPD. Total hospitalization costs were transformed to US dollars using the average exchange rate in 2017 (one US dollar was equivalent to 6.75 yuan).

### Statistical analysis

Categorical data are expressed as percentages, and the chi-square test was used for comparisons between male and female groups. After the normal distribution test, the normally distributed data are expressed as the “mean ± standard deviation,” with a t test used for comparisons, while the abnormally distributed data are expressed as median values with interquartile ranges with a nonparametric test applied to carry out comparisons between the two groups. We additionally compared the in-hospital outcomes and 3-year mortality between female and male smokers with AECOPD. Kaplan-Meier survival analysis and log-rank test were used to describe and compare 3-year mortality between male and female patients in the overall cohort and in smokers. Two-tailed *P* < 0.05 was deemed statistically significant. All analyses were conducted using SPSS version 25.0.

## Results

### Baseline clinical characteristics

**Fig. 1:**
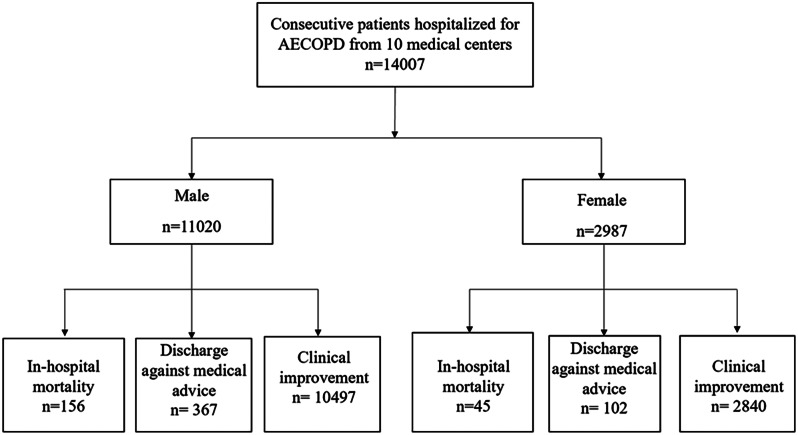
Flow chart of the study

In total, 14,007 patients with AECOPD were included in the MAGNET AECOPD study. The mean age of all study patients was 72.32 ± 10.39 years, and 11,020 (78.7%) were male (Fig. 1). The baseline characteristics and comorbidities of the study patients according to sex are shown in Table 1. Male patients were younger and had a lower BMI than female patients. As expected, smokers were more frequently observed in male patients than in female patients and male smokers had more pack-years of smoking history. There was no significant difference in the frequency of hospitalization due to AECOPD in the past year between male and female patients. Regarding pulmonary function, female patients tended to have a higher FEV_1_/FVC ratio and FEV_1_% predicted. The most common complications in the overall cohort were hypertension, chronic pulmonary heart disease, diabetes, and heart failure. There were significant differences in the prevalence of comorbidities, including heart failure, arrhythmia, bronchiectasis, asthma, chronic pulmonary heart disease, hypertension, diabetes, chronic renal insufficiency, venous thromboembolism, and anxiety-depression, between the two groups, which were more frequently observed in female patients than in male patients (all *P* < 0.05). Pulmonary tuberculosis and active cancer were more prevalent in males. There were no significant differences in the prevalence of coronary heart disease, heart valve problems, interstitial lung disease (ILD), obstructive sleep apnea hypopnea syndrome (OSAHS) or stroke between the male and female patients. On average, females showed more numerous comorbidities per patient than males (2.22 ± 1.64 vs. 1.73 ± 1.56, *P* < 0.001).

**Table 1:** Baseline characteristics and comorbidities of the study patients according to sex

Variable	Total (N = 14,007)	Male (N = 11,020)	Female (N = 2987)	P Value
Age (median, IQR)	72.32 ± 10.39	71.86 ± 10.23	74.02 ± 10.79	< 0.001
BMI (kg/m^2^)	21.58 ± 4.07	21.38 ± 3.88	22.44 ± 4.69	< 0.001
BMI<18(kg/m^2^)	1517(19.4)	1270(20.0)	247(17.0)	0.009
Smoker( n, %)	8652(61.8)	8351(75.8)	301(10.1)	< 0.001
Pack-years	39.44 ± 26.57	39.92 ± 26.64	25.31 ± 19.76	< 0.001
Current smoker (n, %)	2738(19.6)	2632(23.9)	106(3.6)	< 0.001
Former smoker (n, %)	5914(42.7)	5719(52.4)	195(6.6)	< 0.001
Frequency of hospitalization due to AECOPD in the past year (times), (n, %)				0.284
<2	4221(71.0)	3440(70.7)	781(72.3)	
≥ 2	1726(29.0)	1427(29.3)	299(27.7)	
FEV_1_/FVC (%)	52.65 ± 13.45	51.00 ± 13.62	57.27 ± 11.82	< 0.001
FEV_1_/% predicted (%)	48.19 ± 23.85	44.72 ± 21.77	59.03 ± 26.80	< 0.001
**Comorbidities (n, %)**				
Coronary heart disease	1546(11.0)	1219(11.1)	327(10.9)	0.860
Heart failure	1609(11.5)	1153(10.5)	456(15.3)	< 0.001
Heart valve problems	250(1.8)	186(1.7)	64(2.1)	0.096
Arrhythmia	1187(8.5)	871(7.9)	316(10.6)	< 0.001
Pulmonary tuberculosis	635(4.5)	564(5.1)	71(2.4)	< 0.001
Bronchiectasis	1198(8.6)	815(7.4)	383(12.8)	< 0.001
ILD	403(2.9)	326(3.0)	77(2.6)	0.270
Asthma	462(3.3)	286(2.6)	176(5.9)	< 0.001
OSAHS	84(0.6)	62(0.6)	22(0.7)	0.275
Chronic pulmonary heart disease	2905(20.7)	2092(19.0)	813(27.2)	< 0.001
Hypertension	4679(33.4)	3514(31.9)	1165(39.0)	< 0.001
Diabetes	1804(12.9)	1306(11.9)	498(16.7)	< 0.001
Stroke	784(5.6)	615(5.6)	169(5.7)	0.871
Venous thromboembolism	520(3.7)	359(3.3)	161(5.4)	< 0.001
Active cancer	800(5.7)	667(6.1)	133(4.5)	0.001
Chronic renal insufficiency	514(3.7)	376(3.4)	138(4.6)	0.002
Anxiety-depression	106(0.8)	69(0.6)	37(1.2)	0.001
No. of comorbidities	1.84 ± 1.60	1.73 ± 1.57	2.23 ± 1.65	< 0.001

*Abbreviations* BMI = body mass index; ILD = interstitial lung disease; OSAHS = Obstructive sleep apnea-hypopnea syndrome

### Clinical manifestations (symptoms and signs)

The most commonly observed clinical manifestations of AECOPD were cough (94.3%), followed by expectoration (91.7%), dyspnea (61.0%), and wheeze (24.6%) (Table 2). Altered mental status, leg edema and P2 hyperactivity were more prevalent in females, and cough and expectoration were more prevalent in males. Regarding vital signs on admission, female patients tended to have lower diastolic blood pressure (DBP) and slower pulse rate (all *P* < 0.001). There was no significant difference in systolic blood pressure (SBP) between the two groups. In males, blood oxygen saturation was significantly lower than that in females (*P* < 0.05).

**Table 2:** Clinical features of the study patients according to sex

Variable	Total (N = 14,007)	Male (N = 11,020)	Female (N = 2987)	P Value
**Symptoms (n, %)**				
Altered mental status	465(3.3)	316(2.9)	149(5.0)	< 0.001
Cough	13,208(94.3)	10,439(94.7)	2769(92.7)	< 0.001
Expectoration	12,848(91.7)	10,178(92.4)	2670(89.4)	< 0.001
Wheeze	3450(24.6)	2693(24.4)	757(25.3)	0.308
Dyspnea	8550(61.0)	6772(61.5)	1778(59.5)	0.055
Fever	1613(11.5)	1288(11.7)	325(10.9)	0.220
**Signs**				
SBP (mmHg)	132.05 ± 19.44	131.91 ± 19.40	132.59 ± 19.57	0.088
SBP < 100 mmHg	552(3.9)	429(3.9)	123(4.1)	0.572
DBP (mmHg)	78.83 ± 12.58	79.04 ± 12.47	78.04 ± 12.96	< 0.001
DBP < 60 mmHg	806(5.8)	584(5.3)	222(7.4)	< 0.001
Pulse rate(bpm)	89.13 ± 16.63	89.76 ± 16.66	86.81 ± 16.31	< 0.001
Pulse rate > 100 bpm	3501(25.0)	2889(26.2)	612(20.5)	< 0.001
Respiratory rate > 30 bpm	91(0.7)	72(0.7)	19(0.6)	0.917
SO_2_(%)	96.7(95.0-98.1)	96.40(95.00–98.00)	97.00(95.18–98.60)	0.006
P2 hyperactivity (n, %)	194(1.4)	138(1.3)	56(1.9)	0.010
Edema of the lower extremities (n, %)	1755(12.5)	1213(11.0)	542(18.1)	< 0.001

*Abbreviations* SBP = Systolic Blood Pressure; DBP = Diastolic blood pressure

### Laboratory and radiographic findings

The laboratory and radiographic findings of the study patients are summarized in Table 3. Anemia and increased white blood cell (WBC) counts were more common in males, whereas decreased eosinophil ratios (EOSRs) and platelet (PLT) counts were more common in females (all *P* < 0.05). Compared to female patients, male patients had higher levels of creatinine and C-reactive protein and had lower levels of albumin in blood. There were no significant differences between the male and female groups in terms of neutrophil ratio (NEUT%), partial pressure of oxygen (PaO2), partial pressure of carbon dioxide (PaCO2), alanine aminotransferase (ALT), aspartate aminotransferase (AST) and urea nitrogen (BUN) as well as N-terminal pro-B type natriuretic peptide (NT-pro BNP), troponin T, D-dimer and procalcitonin (PCT). In CT findings, pulmonary bulla and pulmonary interstitial fibrosis were more frequently observed in male patients, and pulmonary artery enlargement, pleural effusion, atelectasis/consolidation, and bronchiectasis were more common in female patients.

**Table 3:** Laboratory and imaging findings of the study patients according to sex

Variable	Total (N = 14,007)	Male (N = 11,020)	Female (N = 2987)	P Value
**Laboratory tests**				
Hemoglobin (g/L)	127.28 ± 22.53	128.67 ± 22.54	122.16 ± 21.73	< 0.001
Anemia	3962(28.7)	3233(29.8)	729(24.7)	< 0.001
WBC(×10^9^/L)	7.70(5.89–10.19)	7.78(6.00-10.27)	7.34(5.50–9.90)	< 0.001
WBC>10 × 10^9^/L	3589(26.0)	2871(26.4)	718(24.3)	0.019
NEUT (%)	74.34 ± 12.76	74.38 ± 12.76	74.22 ± 12.77	0.570
EOSR (%)	1.0(0.1–2.6)	1.0(0.1–2.8)	0.7(0.1–2.2)	< 0.001
EOSR ≥ 2%	4490(32.7)	3694(34.3)	796(27.2)	< 0.001
Platelet (×10^9^/L)	192(145–249)	194(146–251)	186(141-242.75)	< 0.001
Platelet < 100 × 10^9^/L	1074(7.8)	817(7.5)	257(8.7)	0.031
PH	7.41(7.38–7.45)	7.41(7.38–7.45)	7.42(7.38–7.46)	< 0.001
PH < 7.30	350(3.9)	272(3.7)	78(4.5)	0.126
PaO2 (mmHg)	83.0(68.1–106.0)	83.35(68.80-106.10)	81.0(65.9–106.0)	0.207
PaCO2 (mmHg)	42.3(36.3–51.1)	42.2(36.3–50.70)	42.60(36.15–53.20)	0.077
ALT(U/L)	17(12–27)	18.00(12.00-27.70)	16(11–24)	0.336
AST(U/L)	22(17.4–29.3)	22.00(17.70-29.35)	22(17–29)	0.351
BUN (mmol/L)	5.70(4.30–7.61)	5.70(4.40–7.65)	5.40(4.00-7.56)	0.231
ALB(g/L)	36.8(33.4–40.0)	36.60(33.30–39.80)	37.40(33.90–40.80)	< 0.001
ALB < 35 g/L	4669(35.3)	3780(36.3)	889(31.8)	< 0.001
Creatinine (µmol/L)	75.65(61.0–96.0)	78.00(64.25-98.00)	65.00(53.00–86.00)	< 0.001
Increased creatinine	2628(19.3)	2098(19.6)	530(18.4)	0.148
NT-pro BNP (pg/ml)	320.00(103.00-1309.50)	283.00(91.91-1132.50)	55.35(169.00-2140.25)	0.994
cTNT (ng/L)	18.00(10.80–33.20)	18.60(11.10–34.50)	16.40(9.70–30.50)	0.104
D-dimer(mg/L)	0.73(0.40–1.54)	0.70(0.39–1.46)	0.87(0.48–1.82)	0.070
PCT (ng/ml)	0.12(0.05–0.43)	0.13(0.05–0.45)	0.10(0.05–0.37)	0.900
CRP (mg/L)	12.40(3.89–47.40)	12.90(3.84–51.40)	11.20(4.07–37.33)	< 0.001
CRP > 10 mg/L	4707(54.7)	3655(55.3)	1052(52.8)	0.052
**Radiological findings**				
Pulmonary artery enlargement	265(1.9)	159(1.4)	106(3.5)	< 0.001
Pleural effusion	2619(18.7)	1993(18.1)	626(21.0)	< 0.001
Pulmonary bulla	3589(25.6)	3199(29.0)	390(13.1)	< 0.001
Atelectasis/consolidation	755(5.4)	537(4.9)	218(7.3)	< 0.001
Bronchiectasis	2021(14.4)	1421(12.9)	600(20.1)	< 0.001
Pulmonary interstitial fibrosis	963(6.9)	789(7.2)	174(5.8)	0.011
Right ventricular enlargement	125(0.9)	42(0.4)	16(0.5)	0.243

*Abbreviations* WBC = white blood cell; NEUT = neutrophil ratio; EOSR = eosinophil ratio; ALT = alanine aminotransferase; AST = aspartate aminotransferase; BUN = blood urea nitrogen; ALB = albumin; NT-proBNP = N-terminal pro-B-type natriuretic peptide; cTNT = cardiac troponin T; PCT = procalcitonin; CRP = C-reactive protein

### Treatments characteristics

During hospitalization, inhaled/nebulized bronchodilators, inhaled/nebulized corticosteroids, systemic corticosteroids, antibiotics and theophylline drugs were given to 70.4%, 56.0%, 41.0%, 64.9% and 55.1% of the overall cohort, respectively (Table 4). In sex comparisons, inhaled/nebulized bronchodilators, inhaled/nebulized corticosteroids, systemic corticosteroids and theophylline drugs were all prescribed more often to male patients than to female patients. Females were more likely to receive antibiotics than males. For respiratory support, 20.1% and 3.3% of the patients received noninvasive and invasive mechanical ventilation during hospitalization, respectively. Females with AECOPD more often received high levels of respiratory support than males (including noninvasive (22.8% vs. 19.3%, *P* < 0.001) or invasive mechanical ventilation (4.3% vs. 3.0%, *P* = 0.001).

**Table 4:** Usage of treatment in the study patients according to sex

Variable	Total (N = 14,007)	Male (N = 11,020)	female (N = 2987)	P
Inhaled/nebulized bronchodilators (beta-adrenoceptor agonist or muscarinic receptor antagonist) (n, %)	9859(70.4)	7925(71.9)	1934(64.7)	< 0.001
Inhaled/nebulized corticosteroids (n, %)	7850(56.0)	6234(56.6)	1616(54.1)	0.016
Oral corticosteroids (n, %)	1500(10.7)	1254(11.4)	246(8.2)	< 0.001
Intravenous corticosteroids (n, %)	4982(35.6)	4095(37.2)	887(29.7)	< 0.001
Systemic corticosteroid (Oral or IV)	5744(41.0)	4722(42.8)	1022(34.2)	< 0.001
Antibiotics (n, %)	9088(64.9)	7023(63.7)	2065(69.1)	< 0.001
Theophylline drugs (n, %)	7719(55.1)	6127(55.6)	1592(53.3)	0.025
Non-invasive ventilation (n, %)	2811(20.1)	2130(19.3)	681(22.8)	< 0.001
Invasive ventilation (n, %)	462(3.3)	334(3.0)	128(4.3)	0.001

### In-hospital and long-term clinical outcomes and sex differences in the overall cohort

**Fig. 2:**
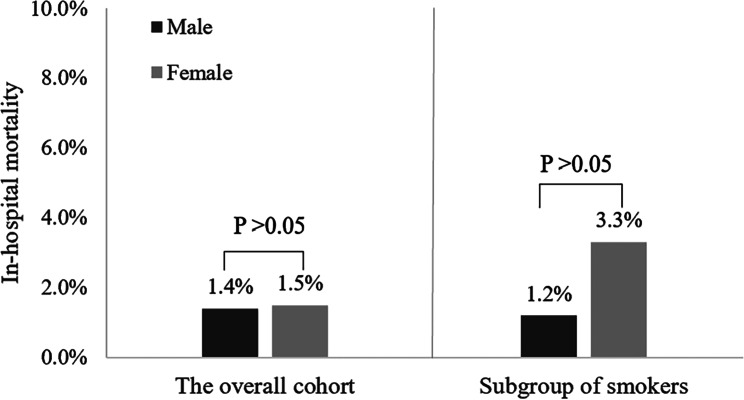
The in-hospital mortality in the overall cohort and the subgroup of smokers according to sex

**Fig. 3:**
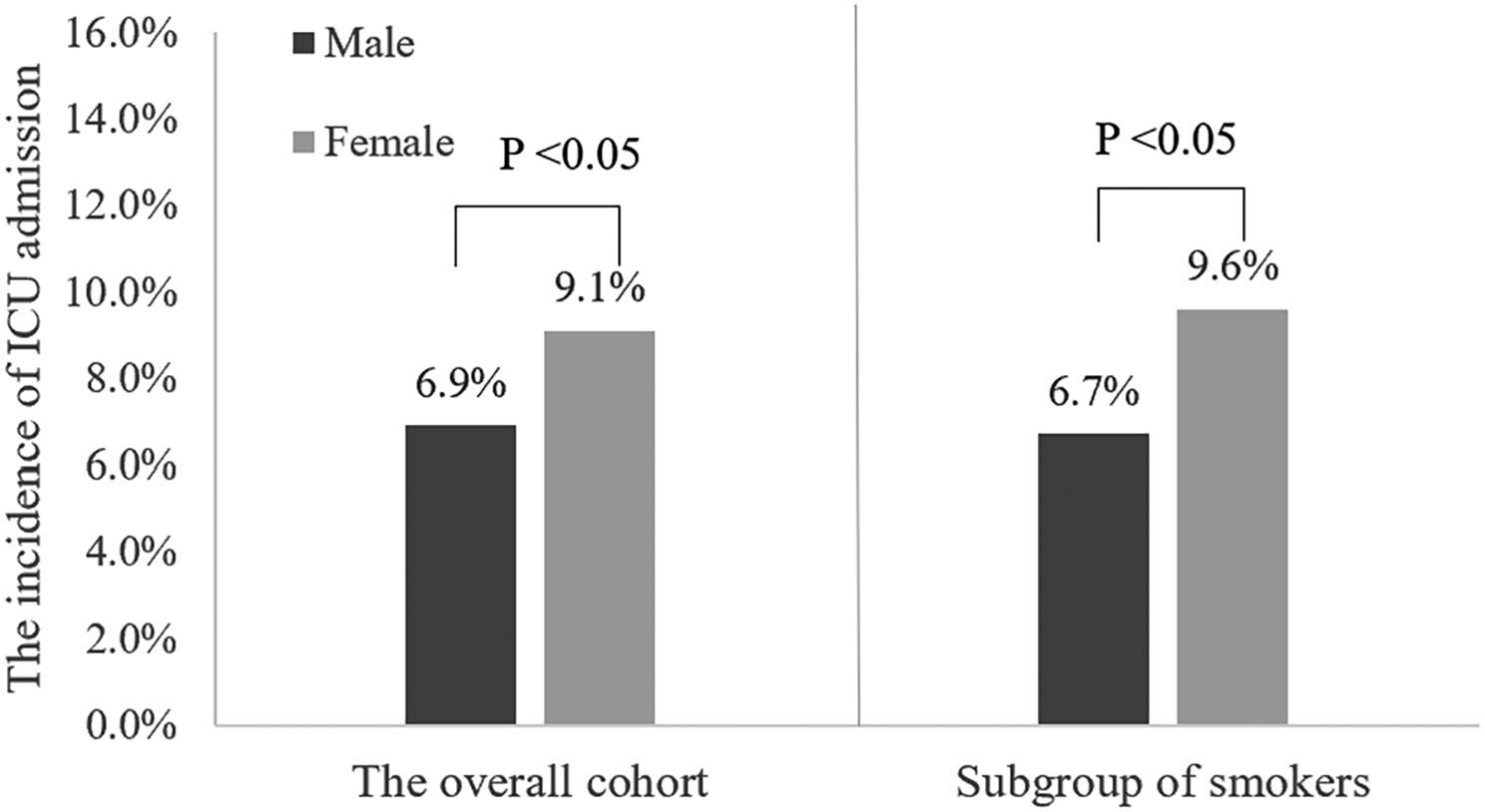
The incidence of ICU admission at discharge in the overall cohort and the subgroup of smokers according to sex

For all patients enrolled in our study, the overall all-cause mortality during hospitalization was 1.4%, which corresponded to 201 cases of death (Table 5); 469 (3.3%) discharged against medical advice, 13,337 (95.2%) had been discharged from hospitals after showing improvement, and 1035 (7.4%) were admitted into the ICU. The median LOS was 9 days, and the total cost was 1911 (IQR: 1294–2936) US dollars. No statistically significant differences in hospital mortality (1.4% vs. 1.5%, *P* = 0.711) and incidence of discharge against medical advice (3.3% vs. 3.4%, *P* = 0.820) were observed between male and female patients with AECOPD (Fig. 2 and Supplementary Fig. 1). ICU admission in female patients was more needed during hospitalization than in male patients (9.1% vs. 6.9%, *P* < 0.001) (Fig. 3). However, the mean length of stay and total costs during hospitalization showed no significant differences between the two groups.

**Table 5:** Short-term and long-term clinical outcomes in the overall cohort of AECOPD inpatients according to sex

Variable	Total (N = 14,007)	Male (N = 11,020)	Female (N = 2987)	P
In-hospital mortality (n, %)	201(1.4)	156(1.4)	45(1.5)	0.711
Discharge against medical advice (n, %)	469(3.3)	367(3.3)	102(3.4)	0.820
Clinical improvement (n, %)	13,337(95.2)	10,497(95.3)	2840(95.1)	0.690
ICU admission (n, %)	1035(7.4)	764(6.9)	271(9.1)	< 0.001
Length of stay (days, median, IQR)	9(6–13)	9(6–13)	10(7–14)	0.549
Total hospitalization expenses	1911(1294–2936)	1976(1350–3009)	1631(1049–2647)	0.394
3-year mortality (n, %) ^a^	978(34.3)	752(35.3)	226(31.4)	0.058

*Abbreviations* ICU = intensive care unit

^a^ Available in 2852 patients

**Fig. 4:**
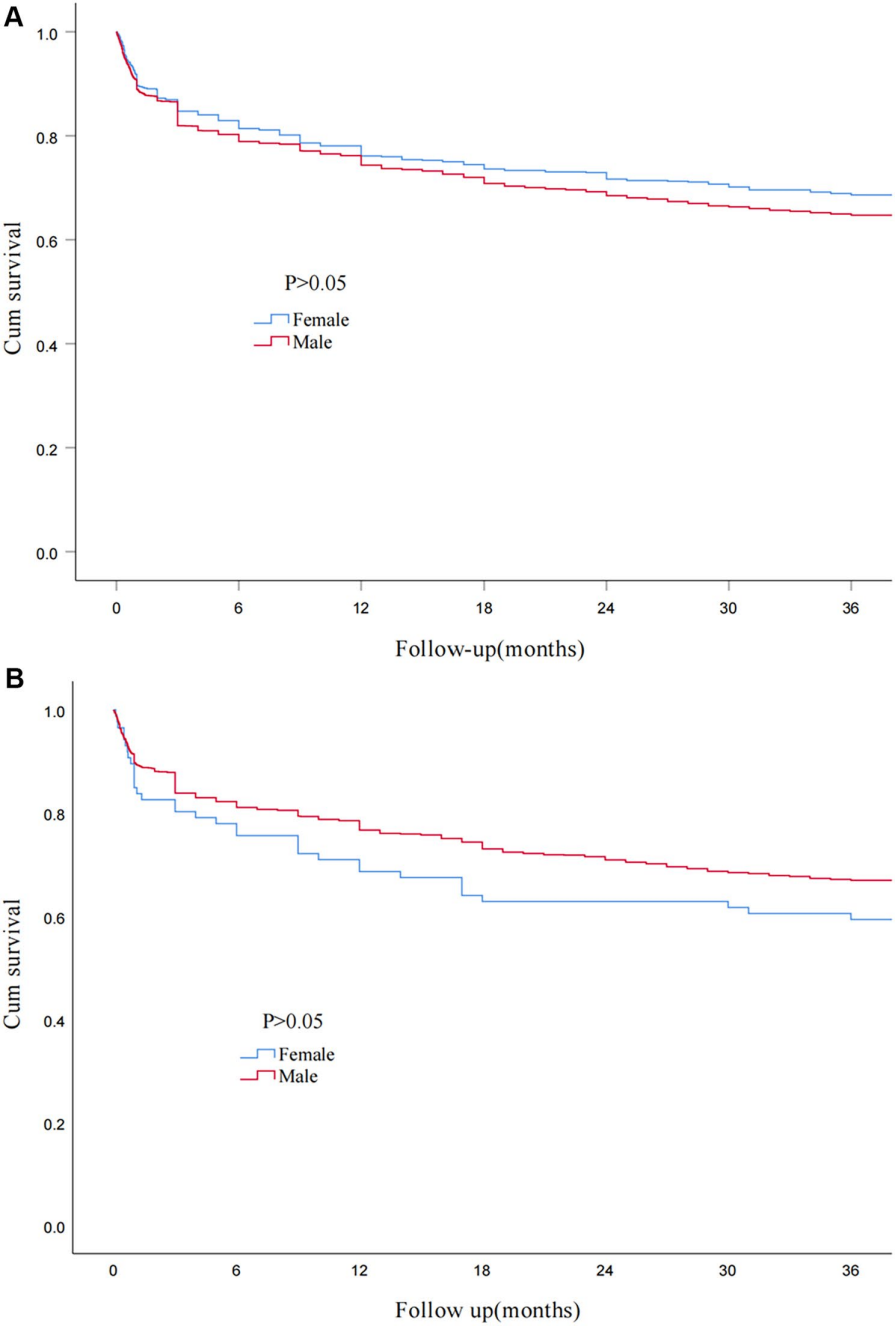
(**A**) Survival curve of the overall cohort according to sex. (**B**) Survival curve of the subgroup of smokers according to sex

A total of 2852 patients from two out of the ten medical centers were followed up for 3 years, 978 patients (752 males and 226 females) died during the follow-up corresponding to a relatively high all-cause mortality of 34.3% (Supplementary Fig. 2). There was no significant difference in the 3-year mortality between male and female patients (35.3% vs. 31.4%, *P* = 0.058), although a higher trend in males can be observed. Using Kaplan-Meier survival analysis, the 3-year survival difference between male and female patients was not found to be significantly different either. (*P* = 0.06; Fig. 4A).

### In-hospital and long-term clinical outcomes and sex differences in smokers

A total of 8652 (61.8%) smokers with AECOPD were included in our study. Among them, 8351 (96.5%) were males, and 301 (3.5%) were females (Supplementary Fig. 3). The in-hospital and long-term clinical outcomes in smokers with AECOPD are shown in Table 6. A total of 112 smokers (1.3%) died during hospitalization, 306 (3.5%) discharged against medical advice, 8234 (95.2%) had been discharged from hospitals after showing improvement, and 586 (6.8%) were admitted into the ICU. Both the in-hospital mortality and incidence of ICU admission in female smokers were significantly higher than those in male smokers (3.3% vs. 1.2%, *P* = 0.002; 9.6% vs. 6.7%, *P* = 0.044) (Figs. 2 and 3). However, the incidence of discharge against medical advice (3.5% vs. 4.0%, *P* = 0.667) showed no significant differences between male and female smokers (Supplementary Fig. 1). The 3-year mortality in female smokers was higher than male smokers, but the difference was not statistically significant (40.7% vs. 33.1%, *P* = 0.146). Similarly, there was no difference in survival between female and male smokers using Kaplan-Meier survival analysis although females showed a trend towards higher long-term mortality (*P* = 0.12; Fig. 4B).

**Table 6:** Short-term and long-term clinical outcomes in smokers with AECOPD according to sex

Variable	Total (N = 8652)	Male (N = 8351)	Female (N = 301)	P
In-hospital mortality (n, %)	112(1.3)	102(1.2)	10(3.3)	0.002
Discharge against medical advice (n, %)	306(3.5)	294(3.5)	12(4.0)	0.667
Clinical improvement (n, %)	8234(95.2)	7955(95.3)	279(92.7)	0.041
ICU admission (n, %)	586(6.8)	557(6.7)	29(9.6)	0.044
Length of stay (days, 9(6–13) 9(6–13) 9(7–14) 0.330 median, IQR)				
Total hospitalization expenses	1986(1395–2987)	1988(1395–2986)	1911(1306–3066)	0.864
3-year mortality (n, %) ^a^	561(33.5)	526(33.1)	35(40.7)	0.146

*Abbreviations* ICU = intensive care unit

^a^ Available in 2852 patients

## Discussion

Numerous studies have described the clinical features, management and prognosis of patients with stable COPD [12, 13, 20, 21]; however, there are limited available data regarding the characteristics, treatments and in-hospital clinical outcomes among AECOPD patients, especially in China. In our multicenter cohort study that enrolled 14,007 patients with AECOPD, the most common complications in the overall cohort were hypertension, chronic pulmonary heart disease, diabetes and heart failure, and the most commonly observed clinical manifestations of AECOPD were cough, followed by expectoration, dyspnea and wheeze, as expected. During hospitalization, inhaled/nebulized bronchodilators, inhaled/nebulized corticosteroids, systemic corticosteroids, antibiotics and theophylline drugs were given to 70.4%, 56.0%, 41.0%, 64.9% and 55.1% of the overall cohort, respectively. In our study, the overall all-cause mortality, the incidence of discharge against medical advice and ICU admission among the population were 1.4%, 3.3% and 7.4%, respectively. The all-cause mortality was lower than previous findings by investigations conducted in the US and Italy [20, 21]. However, after considering the 469 (3.3%) patients discharged against medical advice in most cases because of clinical deterioration, in whom death probably occurred shortly after discharge, the incidence of adverse outcomes was close to those in previous studies [20, 21]. Additionally, a large multicenter investigation recently conducted in China (ACURE study) reported that the overall rate of mortality was only 0.1% in AECOPD inpatients [22]. Compared with the ACURE study, more patients were included in our study (14,007 vs. 5334), although the sex proportion was almost the same in the two studies. However, the median age was older, and the incidence of noninvasive ventilation, invasive ventilation and ICU admission were more common in our cohort than in the ACURE study.

A growing body of evidence suggests that sex differences exist in clinical manifestations and prognosis in stable COPD [23, 24]. However, much less is known about any sex differences in characteristics, treatments and in-hospital clinical outcomes during AECOPD. Therefore, we yielded evidence on the sex differences in clinical characteristics, management patterns and in-hospital and long-term outcomes in the real-life clinical practice of 14,007 AECOPD patients in China, which may contribute to providing better disease management strategies. In summary, there was no significant difference in in-hospital and long-term mortality rate between male and female patients in the overall cohort, despite different clinical characteristics and treatments being observed between the two groups. Interestingly, we found that the in-hospital mortality in female smokers with AECOPD were much higher than that in male smokers (3.3% vs. 1.2%, *P* < 0.05). The sex differences in patients with AECOPD varied among countries and regions. In the United States, females had higher rates of asthma and several psychiatric comorbidities, shorter length of stay and lower 30-day readmission rate [25]. In Spain, muscle damage was more often present in female patients compared to male patients with AECOPD [26]. This discrepancy is probably due to the ethnic difference, or socio-environmental factors.

Although male patients were younger, they tended to have worse nutritional conditions, which was manifested by lower BMI and lower albumin levels. In our study, smokers were more common in males than in females (75.8% vs. 10.1%, *P* < 0.001), and being consistent with this, lung function parameters (FEV_1_/FVC and FEV_1_/% predicted) from stable stage of male patients was worse than those of female patients. This reflected the enormous adverse effect of smoking on lung function [27]. Comorbidities have been shown to impair quality of life and contribute to hospital admissions and mortality in COPD patients [28]. Many researchers have studied the sex differences in comorbidities among COPD patients (most were stable COPD); however, a consensus was not reached, which might be attributed to different study populations in those studies [29–31]. For example, Agusti et al. found that cardiovascular comorbidity and diabetes were less common among females in the ECLIPSE cohort [29]; Almagro et al. acknowledged that females with COPD had less ischemic heart disease and alcoholism but more chronic heart failure and diabetes in a European study [30]. In the current study, we found significant differences in the prevalence of comorbidities between female and male patients. On average, females showed more numerous comorbidities than males, which was similar to the findings of a prospective investigation conducted in Poland [32]. In particular, disease distribution showed that hypertension, heart failure, arrhythmia, bronchiectasis, asthma, chronic pulmonary heart disease, diabetes, chronic renal disease, venous thromboembolism and anxiety-depression were more frequent in females, but malignancy and pulmonary tuberculosis were more frequent in males.

In terms of the clinical manifestations, altered mental status and lower diastolic blood pressure on admission were more prevalent in females. Male patients with AECOPD had higher level of inflammation indices, such as higher white blood cells and C-reactive protein (CRP), but female patients were more likely to have CT manifestations that indicate pulmonary infection, such as atelectasis/consolidation, pleural effusion, bronchiectasis, etc. Eosinophils have become an important biomarker in the management of stable COPD and AECOPD [33, 34]. Cumulative researches have shown that eosinophil counts could guide the use of corticosteroids, and COPD or AECOPD patients with high eosinophils responded better to corticosteroids [35]. In this study, we found that males had a higher eosinophil ratio than females. In terms of management of AECOPD in this real-world study, drug administration including inhaled/nebulized bronchodilators, inhaled/nebulized corticosteroids, systemic corticosteroids and theophylline drugs were more frequent in males, while antibiotics were more frequently provided to females. Similar to our results, Bade et al. considered that female veterans with AECOPD were less likely to be treated with antimuscarinic or combined bronchodilator/inhaled corticosteroid inhaler [25]. It can be inferred that the medication strategies for AECOPD patients basically corresponded to the characteristics of clinical symptoms and laboratory and radiologic results of male and female patients. Compared with females, male patients had more obvious symptoms of cough, expectoration, dyspnea and higher proportion of eosinophils, so as to be more likely to accept bronchodilators and corticosteroids. However, although male patients had higher level of blood inflammation indices, antibiotics were more frequently provided to females. This may suggest that physicians tended to prescribe antibiotics to patients with lung infection indicated by radiologic findings rather than only indicated by blood inflammation indicators in the real world. In addition to with more comorbidities, being more likely to have altered mental status and lower diastolic blood pressure, female patients also had higher incidence of receiving noninvasive and invasive mechanical ventilation than males, which all suggest that female patients with AECOPD may have more serious illness and worse prognosis.

The differences between males and females in the outcomes of AECOPD have not been clearly identified. In a cohort from the Québec provincial health insurance plan in Canada, Gonzalez et al. observed that male sex was associated with a significantly increased risk of mortality and with a significantly increased risk of rehospitalization for obstructive airways disease [36]. In contrast, Machado et al. believed that females with severe COPD had a greater risk of death than male patients [37]. In our study, no statistically significant differences in hospital and long-term mortality were observed between male and female patients with AECOPD, although female patients were more likely to admitted into ICU during hospitalization. Aryal et al. proposed a possibility that females were less likely to self-medicate with anticholinergic agents and were less likely to seek emergency care within the first 24 h of exacerbation, which could at least in part play a role in more serious illness state on admission and higher incidence of ICU admission [38]. Unfortunately, this proposal is hard to confirm in our study. Interestingly, in the subgroup of smokers, we found the in-hospital mortality in female patients was much higher than that in males. There were two main explanations for this finding. First, females may be more susceptible to the harmful effects of smoking than males, leading to more severe disease for the equivalent quantity of cigarettes consumed [39–41]. This perception has been validated in animal studies and human pathology specimens, which have demonstrated a greater burden of small airway disease in females than in males with COPD despite a similar history of tobacco smoke exposure [42, 43]. Second, females are found to quit smoking less frequently and have a lower success rate of long-term smoking cessation than male patients [38], so the long-term tobacco exposure may also aggravates the disease. Being consistent with this explanation, the smoking cessation rate in female smokers was lower than that in male smokers in our study (6.6% vs. 52.4%, *P* < 0.001).

Our study had several strengths including: (a) We elucidated clear and comprehensive sex differences in the characteristics, treatments and in-hospital clinical outcomes of AECOPD using a large number of AECOPD patients from the prospective multicenter registry study. To our knowledge, this was the first large-scale study to explore sex differences in patients with AECOPD in China. (b) Our findings may provide necessary information to guide intervention planning as a way to minimize the burden caused by AECOPD in the Chinese population (for example, we should strengthen propaganda of smoking cessation among female patients, and pay attention to the changes in the condition of female smokers with AECOPD to reduce the adverse prognosis). This study also has several limitations. First, we were unable to assess the GOLD ABCD classifications among patients because the degree of dyspnea was not measured quantitatively. Second, only a small proportion of patients have completed follow-up in our study, which may prevent us from evaluating the sex differences in the long-term clinical outcomes of AECOPD patients without bias.

## Conclusions

Using a large AECOPD cohort, we revealed sex differences in the characteristics, treatments, in-hospital and long-term clinical outcomes of AECOPD. Although there were several sex differences in clinical characteristics and treatments, such as older age, more comorbidities, higher frequency of altered mental status, lower diastolic blood pressure, and receiving higher levels of respiratory support as well as higher incidence of ICU admission in females than in males, there was no sex difference in in-hospital and long-term mortality in patients with AECOPD in the overall cohort. However, the in-hospital mortality in female smokers with AECOPD were much higher than those in male smokers. Our findings may provide necessary information to guide clinical management of AECOPD based on sex differences, and further research is required to understand why potential sex-associated differences exist.

### Electronic supplementary material

Below is the link to the electronic supplementary material.

Supplementary Material 1. Table S1: Variables recorded in MAGNET AECOPD study; Figure S1: The incidence of discharge against medical advice at discharge in the overall cohort and the subgroup of smokers according to sex; Figure S2: The long-term clinical outcomes in patients with AECOPD; Figure S3: The in-hospital clinical outcomes in the smokers with AECOPD

## Data Availability

The data will be shared on reasonable request to the corresponding author.
