# Supplementary material for: Characteristics, treatments, in-hospital and long-term outcomes among inpatients with acute exacerbation of chronic obstructive pulmonary disease in China: sex differences in a large cohort study
Source: BMC Pulm Med. 2024 Mar 11;24:125. doi: 10.1186/s12890-024-02948-4 (PMC10929097; doi:10.1186/s12890-024-02948-4)

| Supplementary Table 1 Variables recorded in MAGNET AECOPD study | |
| --- | --- |
| Baseline characteristics | Age |
|  | Gender |
|  | BMI |
|  | Smoking status |
|  | Frequency of hospitalization due to AECOPD in the past year |
|  | FEV_1_/FVC (%) |
|  | FEV_1_/% predicted (%) |
|  | Co-morbidities |
| Clinical features | Symptoms on admission |
|  | Signs on admission |
| Laboratory and imaging findings | Blood tests |
|  | Radiological abnormalities |
| Treatment | Pharmacologic management |
|  | Mechanical ventilation |
| In-hospital clinical outcomes | In-hospital mortality |
|  | Discharge against medical advice |
|  | Clinical improvement |
|  | ICU admission |
|  | Length of stay |
| Long-term clinical outcomes ^a^ | Total hospitalization expenses  3-year all-cause mortality |

Abbreviations: BMI=body mass index; ICU=intensive care unit

a Available in 2852 patients.

Supplementary Figure 1 The incidence of discharge against medical advice at discharge in the overall cohort and the subgroup of smokers according to sex


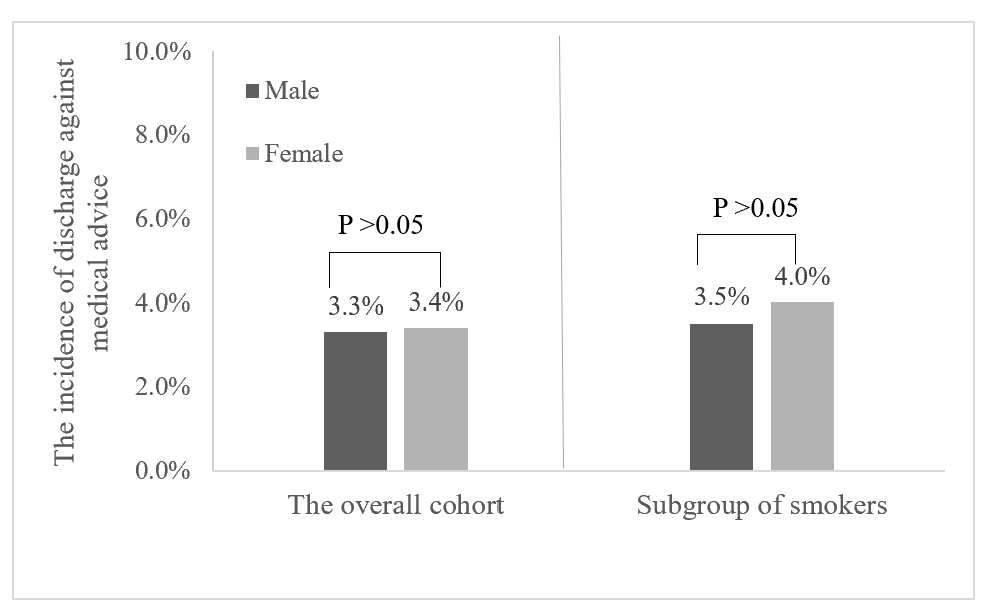


Supplementary Figure 2 The long-term clinical outcomes in patients with AECOPD


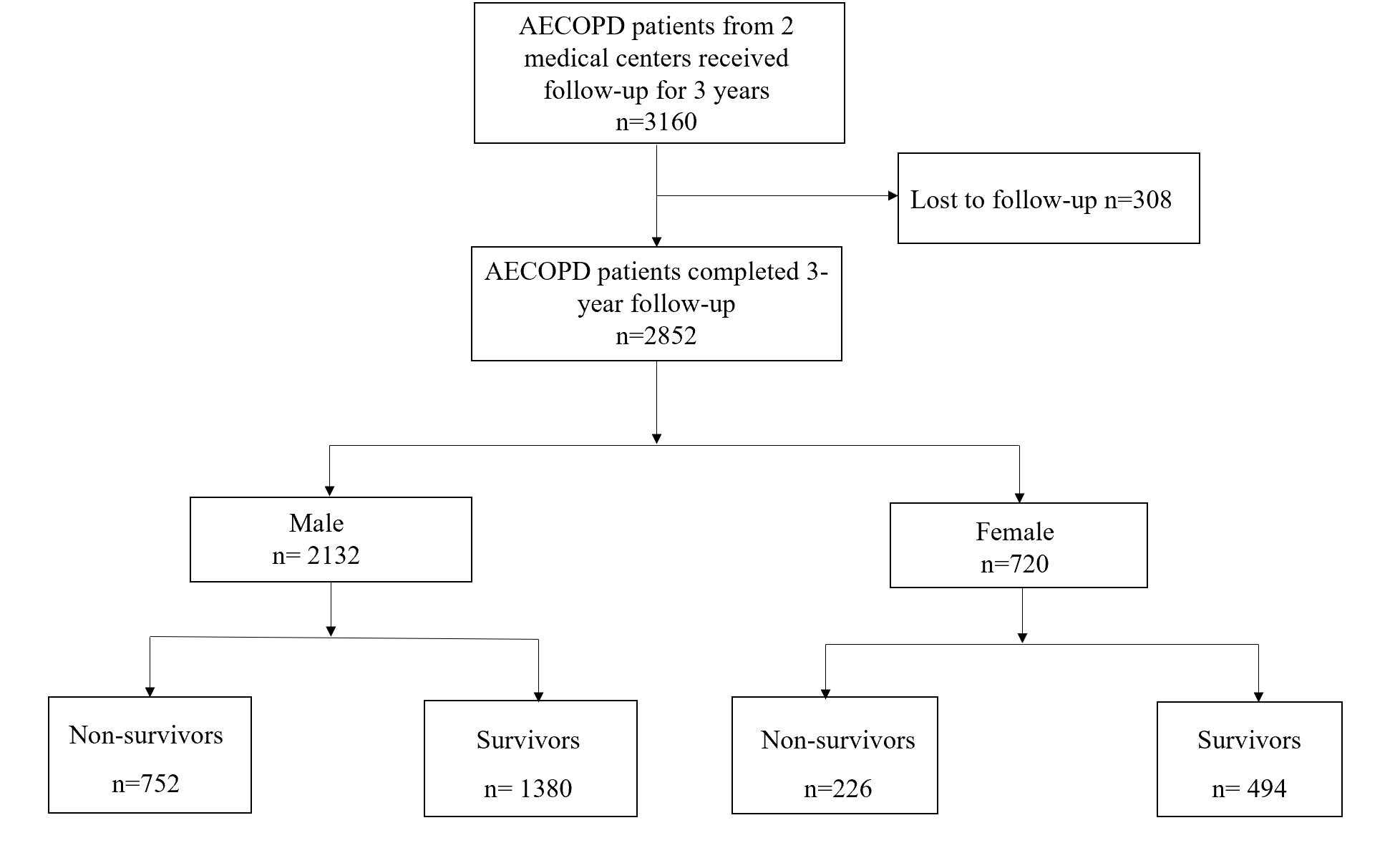


Supplementary Figure 3 The in-hospital clinical outcomes in the smokers with AECOPD


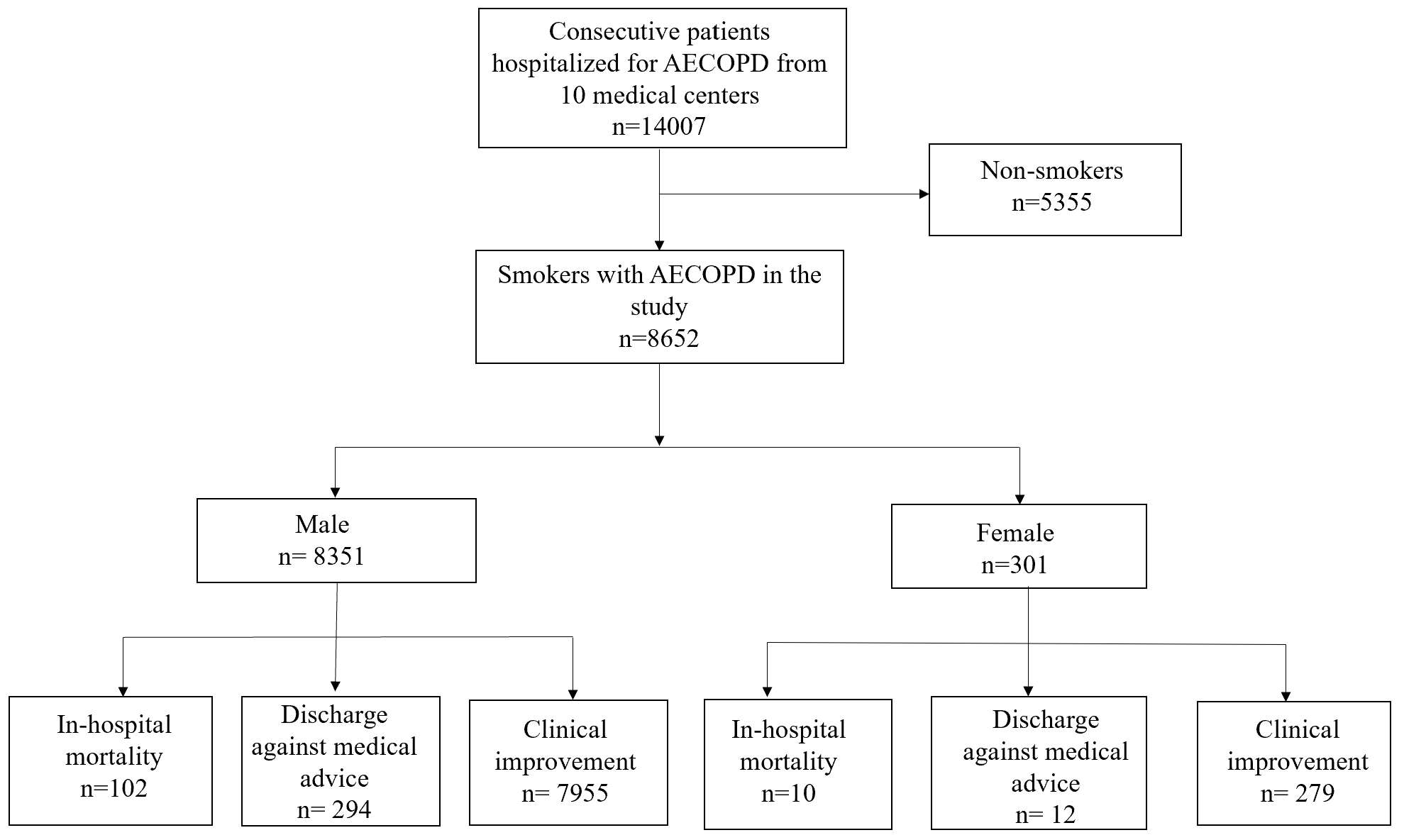

Supplement: Supplementary file 1 — Supplementary Material 1. Table S1: Variables recorded in MAGNET AECOPD study; Figure S1: The incidence of discharge against medical advice at discharge in the overall cohort and the subgroup of smokers according to sex; Figure S2: The long-term clinical outcomes in patients with AECOPD; Figure S3: The in-hospital clinical outcomes in the smokers with AECOPD [file 12890_2024_2948_MOESM1_ESM.docx]
